# Mesenchymal Stromal Cell‐Based Cell–Drug Conjugates for the Treatment of Acute Liver Failure

**DOI:** 10.1002/advs.77411

**Published:** 2026-08-25

**Authors:** Tenghui Ye, Jiamin Wu, Zixin Wu, Xi Liu, Jiajia Luo, Leyi Yang, Peng Shi

**Affiliations:** ^1^ School of Biomedical Sciences and Engineering Guangzhou International Campus South China University of Technology Guangzhou People's Republic of China; ^2^ National Engineering Research Center for Tissue Restoration and Reconstruction South China University of Technology Guangzhou People's Republic of China; ^3^ Guangdong Province Key Laboratory of Biomedical Engineering South China University of Technology Guangzhou People's Republic of China; ^4^ Key Laboratory of Biomedical Materials of the Ministry of Education South China University of Technology Guangzhou People's Republic of China; ^5^ Department of General Surgery Guangzhou First People's Hospital School of Medicine South China University of Technology Guangzhou People's Republic of China

**Keywords:** acute liver failure, cell–drug conjugates, macrophage polarization, mesenchymal stromal cells, rosiglitazone

## Abstract

Acute liver failure (ALF) is a life‐threatening syndrome characterized by severe inflammatory injury and high mortality. Mesenchymal stromal cells (MSCs) hold therapeutic promise for ALF because of their immunomodulatory and tissue‐repair capacities. However, in the ALF inflammatory microenvironment, characterized by abundant activated M1 macrophages and elevated pro‐inflammatory cytokines, MSCs are susceptible to apoptosis, resulting in low survival and limited therapeutic efficacy. Here, we combined the anti‐inflammatory small‐molecule rosiglitazone with MSCs to construct a cell–drug conjugate (CDC) using MSCs as cellular carriers. Rosiglitazone was encapsulated within nanoparticles coated with neutrophil membranes and anchored onto the MSC surface. Sustained release of rosiglitazone from the MSC surface intrinsically enhanced MSC proliferation and paracrine activity through PI3K–Akt pathway activation and extrinsically modulated the inflammatory microenvironment by promoting macrophage polarization toward an anti‐inflammatory phenotype. In a mouse model of ALF, rosiglitazone nanoparticle‐modified MSCs exhibited enhanced accumulation and prolonged retention in the injured liver, more effectively inhibited hepatocyte apoptosis and promoted hepatocyte proliferation, thereby achieving superior therapeutic efficacy. Thus, our study provides a promising strategy for enhancing MSC‐based therapy in ALF.

## Graphical abstract

Mesenchymal stromal cell (MSC)‐based cell‐drug conjugates are constructed by anchoring rosiglitazone‐loaded nanoparticles onto the MSC surface. Sustained rosiglitazone release enhances MSC proliferation and paracrine activity while promoting anti‐inflammatory macrophage polarization. This strategy combines the anti‐inflammatory activity of rosiglitazone with the tissue‐repair capacity of MSCs, resulting in superior therapeutic efficacy in acute liver failure.

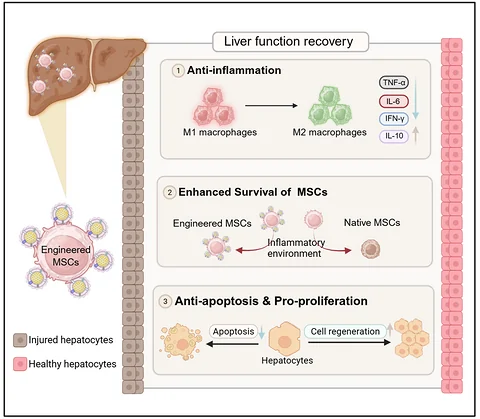

## 1 Introduction

Acute liver failure (ALF) is a life‐threatening syndrome characterized by rapid hepatic dysfunction and high mortality [1, 2, 3]. Current drug therapies show limited efficacy because of poor targeting, adverse effects, and inadequate suppression of acute inflammation [4]. Liver transplantation remains the most effective treatment, but its application is restricted by donor shortage, immune rejection, and surgical complexity [5, 6, 7, 8]. Consequently, the development of safe and effective therapeutic strategies that can serve as alternatives to liver transplantation is of critical importance for improving the overall management of ALF [9, 10]. In recent years, mesenchymal stromal cells (MSCs) have emerged as a promising therapeutic candidate for ALF owing to their potent immunomodulatory, anti‐inflammatory, and tissue‐repairing properties [11, 12], demonstrating considerable potential in both preclinical studies and clinical trials [13, 14, 15].

MSCs possess the ability to actively migrate toward sites of tissue injury and contribute to tissue repair in various models of liver injury by secreting a broad range of bioactive factors [16], suppressing excessive immune responses, and promoting hepatocyte regeneration [17, 18]. Clinical studies have demonstrated the therapeutic potential and preliminary efficacy of MSC infusion for ALF, although its therapeutic outcomes remain unstable [19]. One important reason is that, within the highly inflammatory microenvironment characteristic of ALF, MSCs often show limited survival, thereby compromising their overall therapeutic efficacy [20]. Accumulating evidence indicates that inflammation‐activated macrophages with a pro‐inflammatory phenotype continuously release cytokines such as TNF‐α and IL‐6, which not only exacerbate hepatic tissue damage but also induce apoptosis of transplanted MSCs, thereby significantly compromising their therapeutic potential [21, 22, 23]. Therefore, we propose combining macrophage polarization‐modulating drugs with MSCs for ALF treatment [24]. These drugs can reduce the release of pro‐inflammatory cytokines by modulating macrophage polarization and alleviating the inflammatory microenvironment, which is also favorable for the survival of transplanted MSCs, thereby contributing to a synergistic therapeutic effect [25].

**FIGURE 1:**
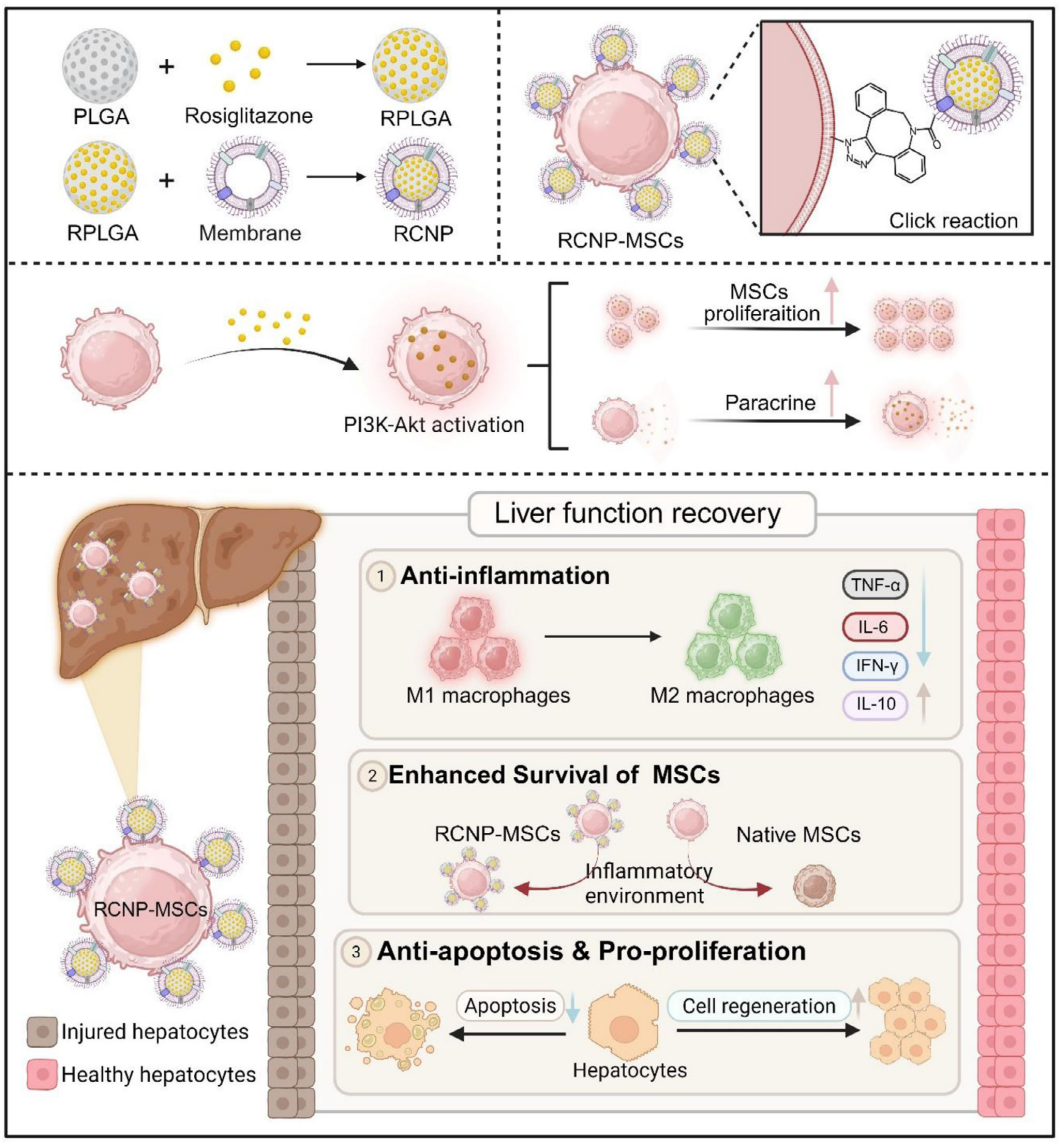
Schematic illustration of the construction of RCNP‐MSCs and their application for the treatment of ALF. RPLGA, rosiglitazone‐loaded PLGA nanoparticles; RCNP, rosiglitazone‐loaded neutrophil‐mimetic nanoparticles; RCNP‐MSCs, MSCs modified with rosiglitazone‐loaded neutrophil‐mimetic nanoparticles.

Cell–drug conjugates (CDCs) provide a novel technological strategy for achieving synergistic integration of pharmacological and cell‐based therapies [26, 27, 28, 29]. In this study, we encapsulated rosiglitazone, a small‐molecule drug capable of promoting the transition of pro‐inflammatory (M1) macrophages toward an anti‐inflammatory (M2) phenotype [30], into nanoparticles and stably anchored them onto the surface of MSCs, thereby constructing MSC‐based CDCs (Figure 1). The modified rosiglitazone enables sustained release on the MSC surface, on the one hand, it enhances MSC proliferation and tissue repair capacity by activating the PI3K–Akt pathway; on the other hand, it promotes the phenotypic transition of surrounding macrophages, reduces pro‐inflammatory cytokine levels, and improves the inflammatory microenvironment, thereby increasing MSC survival under inflammatory conditions. In vivo experiments demonstrated that, in a mouse model of ALF, rosiglitazone‐modified MSCs attenuated inflammation by suppressing pro‐inflammatory responses and promoting macrophage polarization toward an anti‐inflammatory phenotype, exhibited prolonged retention in the injured liver, and facilitated hepatic tissue repair by reducing hepatocyte apoptosis and promoting hepatocyte proliferation. Collectively, this study provides a novel therapeutic strategy for the treatment of ALF and expands the potential applications of cell–drug conjugates in inflammatory diseases.

## 2 Results

### 2.1 Construction of RCNP and RCNP‐MSCs

**FIGURE 2:**
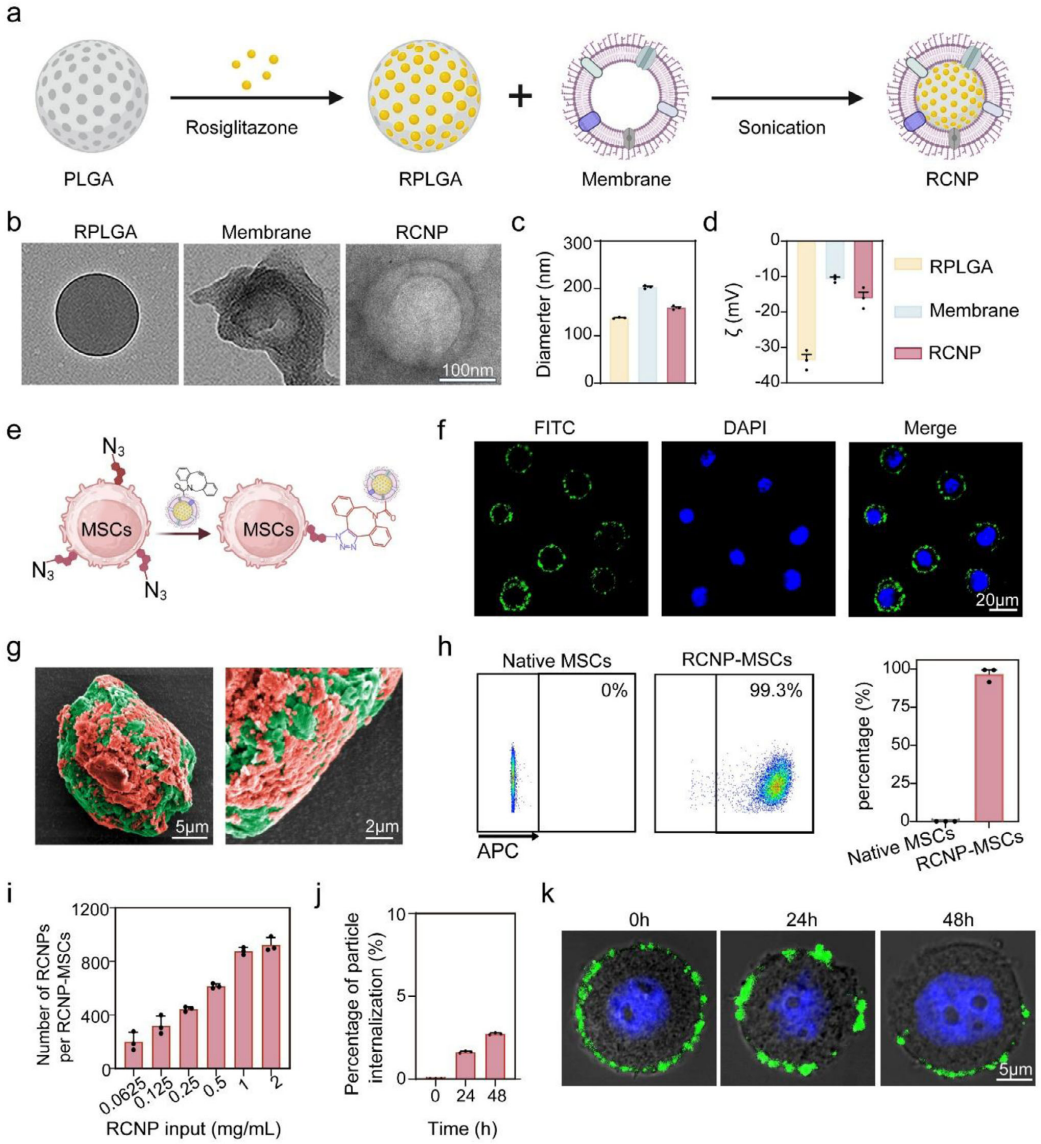
Synthesis and characterization of RCNP and RCNP‐MSCs. (a) Schematic diagram illustrating the synthesis of RCNP. RCNP is constructed by coating rosiglitazone‐loaded PLGA cores with cell membranes. (b) Representative TEM images of RPLGA, Membrane (cell membrane vesicles), and RCNP. (c,d) Size (c) and surface zeta potential (d) of RCNP, Membrane, and RPLGA. Means ± SEM, n = 3 independent experiments. (e) Schematic illustration of the conjugation process of RCNP onto the MSC surface. (f) Confocal fluorescence image of RCNP‐MSCs. Green: DiO‐labeled RCNP; Blue: Hoechst 33342‐labeled cell nuclei. (g) Representative pseudo‐colored SEM image of RCNP‐MSCs, showing MSCs in green and surface‐bound RCNP in red. (h) Flow cytometric analysis (left) and quantification (right) of the surface modification efficiency of MSCs with RCNP. Means ± SEM, n = 3 independent experiments. (i) Quantification of the number of RCNP particles bound per MSC at different initial RCNP concentrations. Means ± SEM, n = 3 independent experiments. (j) Quantification of RCNP internalization by MSCs. Extracellular trypan blue quenching was used to differentiate between surface‐bound and internalized RCNP at 0, 24, and 48 h. Means ± SEM, n = 3 independent experiments. (k) Representative confocal fluorescence images of MSCs immediately after conjugation with fluorescent RCNP (0 h) and after 24 and 48 h of culture. Green: Alexa Fluor 488‐labeled RCNP; Blue: DAPI‐labeled cell nuclei. RPLGA, rosiglitazone‐loaded PLGA nanoparticles; RCNP, rosiglitazone‐loaded neutrophil‐mimetic nanoparticles; Native MSCs, unmodified MSCs; RCNP‐MSCs, MSCs modified with rosiglitazone‐loaded neutrophil‐mimetic nanoparticles.

To improve the in vivo stability and biocompatibility of rosiglitazone [31], we first prepared rosiglitazone‐loaded poly(lactic‐co‐glycolic acid) (PLGA) nanoparticles (RPLGA). Subsequently, RPLGA was coated with neutrophil‐like cell membranes to construct cell membrane‐coated nanoparticles (RCNP) (Figure 2a). Cell membrane coating can endow nanoparticles with source cell‐derived biomimetic properties, such as improved biocompatibility and reduced immune clearance, while also enabling sustained drug release [32, 33]. In particular, previous studies have demonstrated that neutrophil membrane‐coated nanoparticles exhibit enhanced targeting to inflammatory sites by retaining inflammation‐associated membrane proteins involved in neutrophil adhesion and homing [34, 35]. TEM images revealed that RCNP displayed a typical core–shell structure, indicating the successful formation of the cell membrane coating (Figure 2b). Dynamic light scattering (DLS) analysis further showed that membrane coating increased the average hydrodynamic diameter of the nanoparticles from 137.5 ± 1.6 nm to 159.0 ± 4.0 nm (Figure 2c). The polydispersity index (PDI) values of RPLGA, membrane vesicles, and RCNP were 0.038, 0.270, and 0.348, respectively (Figure S1), indicating a broader particle size distribution following membrane coating. Meanwhile, the ζ potential shifted from −33.6 to −16.1 mV (Figure 2d), consistent with the alteration of surface physicochemical properties after membrane coating [35]. We next evaluated the drug loading and encapsulation performance of RCNP. The results showed that RCNP exhibited a drug loading capacity of 4.5 wt.% and an encapsulation efficiency of 18.1% (Figure S2). Notably, the cell membrane coating markedly slowed drug release and conferred RCNP with a more sustained‐release profile. Within 24 h, the cumulative release of rosiglitazone from RCNP was less than 50%, which was significantly lower than that from uncoated RPLGA nanoparticles (Figure S3).

Next, we conjugated RCNP to MSCs via a copper‐free click chemistry reaction [36], thereby constructing RCNP‐modified MSCs (RCNP‐MSCs) (Figure 2e). We first characterized RCNP‐MSCs by confocal fluorescence microscopy, which showed strong green fluorescence on the MSC surface, confirming the successful surface conjugation of RCNP (Figure 2f). Scanning electron microscopy (SEM) further revealed the surface morphology of the engineered MSCs, clearly showing RCNP particles adhering to the cell surface (Figure 2g). Flow cytometric analysis demonstrated that more than 99% of MSCs were successfully loaded with RCNP (Figure 2h), indicating the high efficiency of this conjugation strategy. To optimize the surface loading of RCNP, MSCs were incubated with different concentrations of RCNP, followed by quantitative analysis of nanoparticle binding. The results showed that the amount of RCNP bound to the MSC surface increased with the input concentration and reached saturation at 1 mg/mL (Figure S4a). Based on this result, we further estimated the number of nanoparticles loaded onto each MSC and found that, at 1 mg/mL, each RCNP‐MSC carried an average of approximately 877 RCNP particles (Figure 2i). In addition, calculation of the cell‐associated rosiglitazone content showed that rosiglitazone loading increased with increasing RCNP concentration and plateaued at 1 mg/mL; under this condition, every 10^7^ MSCs carried approximately 1.18 µg of rosiglitazone (Figure S4b).

Subsequently, we evaluated the stability of RCNP modification on the MSC surface. First, we assessed the internalization of surface‐bound RCNP by MSCs. The results showed that during a 48 h incubation period, the internalization rate remained below 5% (Figure 2j and Figure S5), indicating that most nanoparticles remained on the cell surface rather than being internalized. The limited internalization of RCNPs may result from a combination of their stable covalent anchoring through click chemistry, the relatively low phagocytic activity of MSCs, and reduced nonspecific cellular uptake associated with the membrane coating, which together favor particle retention on the cell surface [37, 38, 39]. Furthermore, confocal imaging was used to dynamically monitor the fluorescence signal of RCNP on the MSC surface. The results showed that after 24 h, the fluorescence intensity on the surface of RCNP‐MSCs remained at approximately 50% of its initial level, and after 48 h, more than 24% of the signal was still retained (Figure 2k and Figure S6). Given the minimal internalization observed, we reasoned that the fluorescence decay was mainly attributable to dilution of the membrane‐associated signal during cell proliferation. These results indicate that RCNP modification exhibits good stability on the MSC surface.

Finally, we evaluated the effects of RCNP modification on the in vitro biological functions of MSCs. Live/dead staining showed that RCNP modification did not affect MSC viability, and the cells maintained a healthy growth state (Figure S7a). Migration assays further demonstrated that surface modification did not impair the critical transendothelial migration ability of MSCs (Figure S7b). Together, these findings indicate that RCNP modification preserves the basic biological functions of MSCs and exhibits good biocompatibility.

### 2.2 Effects of Rosiglitazone on MSC Function

**FIGURE 3:**
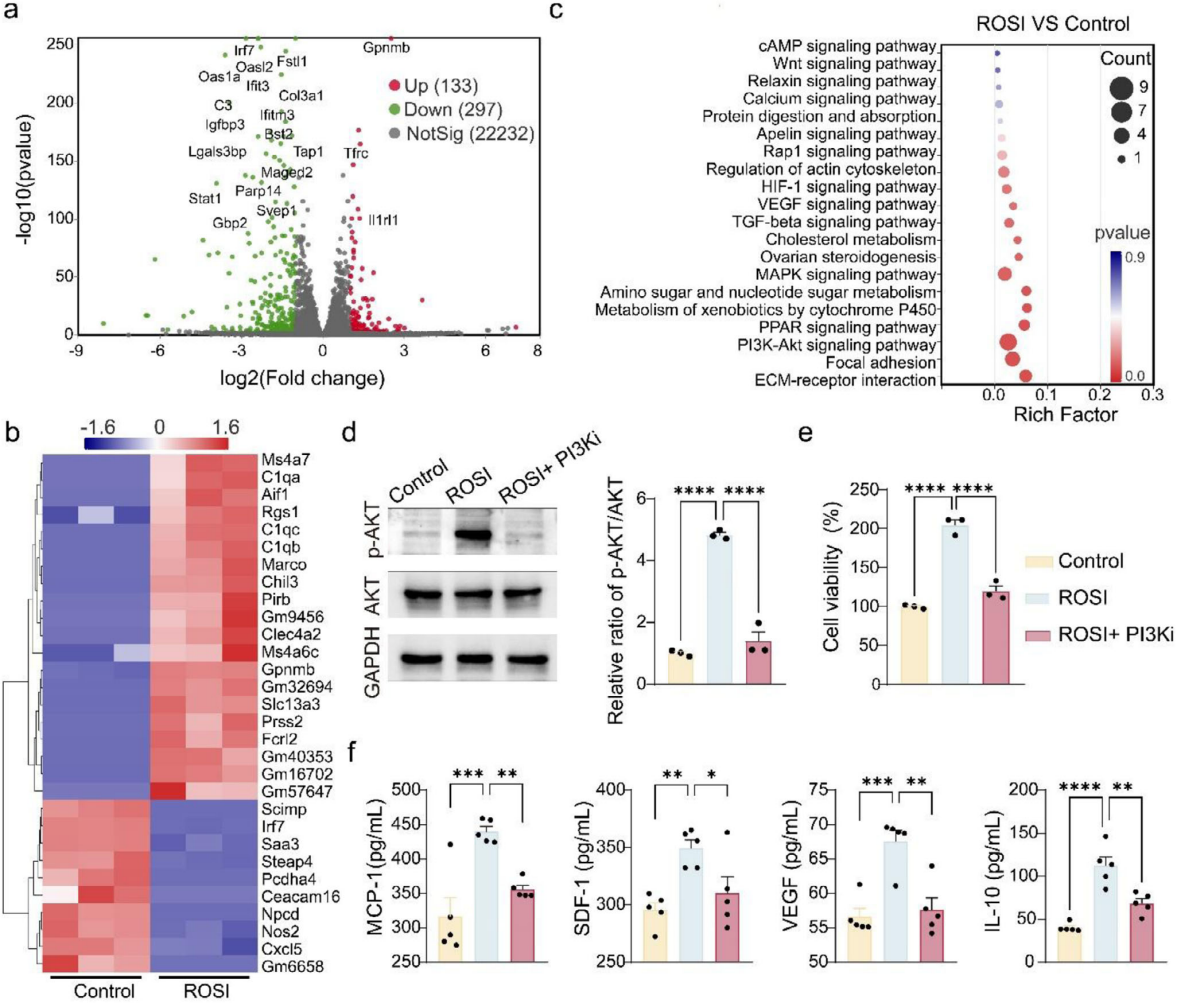
PI3K–Akt signaling contributes to rosiglitazone‐induced enhancement of MSC proliferation and paracrine activity. (a) Volcano plot showing differential gene expression in MSCs treated with rosiglitazone (ROSI) compared to control MSCs. Upregulated (red) and downregulated (green) genes are indicated. (b) Heatmap of gene expression profiling in MSCs treated with ROSI, highlighting key differentially expressed genes. (c) KEGG pathway analysis of differentially upregulated genes in MSCs between the ROSI and Control groups. (d) Western blot analysis of phosphorylated AKT (p‐AKT) and total AKT expression in MSCs with or without PI3K inhibitor treatment (PI3Ki). LY294002 was used as the PI3K inhibitor. Means ± SEM, n = 3 independent experiments. (e) Cell proliferation analysis of MSCs treated with ROSI in the absence or presence of PI3K inhibitor. Means ± SEM, n = 3 independent experiments. (f) Cytokine secretion analysis of conditioned medium from ROSI‐treated MSCs, showing increased secretion of MCP‐1, SDF‐1, VEGF, and IL‐10 compared with control MSCs. Secreted factor levels were normalized to the number of viable cells. Means ± SEM, n = 5 independent experiments. For (d), (e), and (f), statistical analysis was performed by one‐way ANOVA with Tukey's multiple‐comparisons test (^*^ *p* < 0.05; ^**^ *p* < 0.01; ^***^ *p* < 0.001; ^****^ *p* < 0.0001). Control, untreated MSCs; ROSI, rosiglitazone‐treated MSCs.

After successfully encapsulating rosiglitazone in RCNP and conjugating RCNP onto the MSC surface, we first examined whether the surface‐retained drug affected intracellular signaling in MSCs. Specifically, we assessed the expression of PPARγ and the cell cycle‐related gene Cyclin D1 [40]. qPCR results showed that rosiglitazone treatment significantly upregulated the mRNA expression of both PPARγ and Cyclin D1 (Figure S8), consistent with its role as a PPARγ agonist [41, 42]. To systematically evaluate the effects of rosiglitazone on the functional phenotype of MSCs, we further performed RNA sequencing (RNA‐seq) analysis of rosiglitazone‐treated MSCs. Principal component analysis (PCA) showed clear separation between the treated and untreated control groups, with tight clustering of samples within each group, indicating that rosiglitazone induced stable and global transcriptional changes (Figure S9a). Differential expression analysis, visualized by a volcano plot, revealed a large number of significantly upregulated and downregulated genes in the rosiglitazone‐treated group compared with the control group (Figure 3a). Heatmap analysis further demonstrated a clear distinction in global gene expression patterns between the two groups (Figure 3b), suggesting that rosiglitazone markedly reshaped the MSC transcriptome. Among the differentially expressed genes, the upregulated genes were mainly associated with cell cycle, proliferation, and extracellular matrix adhesion‐related processes, whereas the downregulated genes were primarily involved in inflammatory responses and cytokine signaling, indicating that rosiglitazone may enhance MSC proliferation and tissue repair capacity. KEGG pathway enrichment analysis further showed that the upregulated genes were significantly enriched in the PI3K–Akt signaling pathway and ECM–receptor interaction, both of which are closely related to proliferation and tissue repair (Figure 3c). In contrast, the downregulated genes were mainly enriched in inflammation‐ and immune‐related pathways, including TNF and IL‐17 signaling (Figure S9b), suggesting that rosiglitazone may suppress the inflammatory phenotype of MSCs at the transcriptional level [43].

Given that KEGG analysis identified the PI3K–Akt signaling pathway as one of the most significantly upregulated pathways following rosiglitazone treatment, we next examined the involvement of this pathway. Western blot analysis showed that rosiglitazone treatment increased phosphorylated AKT (p‐AKT) levels without altering total AKT expression. This increase in p‐AKT was markedly attenuated by the addition of a PI3K inhibitor (Figure 3d), supporting the activation of PI3K–Akt signaling following rosiglitazone treatment. Consistently, rosiglitazone promoted MSC proliferation in a dose‐dependent manner (Figure S10), whereas this pro‐proliferative effect was markedly attenuated by PI3K inhibition (Figure 3e), suggesting that PI3K–Akt signaling contributes to rosiglitazone‐induced MSC proliferation. In addition, because transcriptomic analysis suggested that rosiglitazone may enhance the tissue repair and immunoregulatory functions of MSCs, we further examined changes in their paracrine profile. ELISA analysis of representative cytokines showed that rosiglitazone treatment significantly increased the secretion of MCP‐1, SDF‐1, VEGF, and IL‐10, whereas the levels of HGF and FGF‐2 were not significantly changed, indicating that rosiglitazone selectively modulates the MSC secretome (Figure 3f and Figure S11). Notably, the enhanced secretion of these factors was significantly attenuated in the presence of the PI3K inhibitor, suggesting that PI3K–Akt signaling also contributes to rosiglitazone‐mediated paracrine regulation. Collectively, these results indicate that activation of the PI3K–Akt signaling pathway plays an important role in mediating the rosiglitazone‐induced enhancement of MSC proliferation and paracrine activity.

### 2.3 Immunomodulatory Effects of RCNP‐MSCs In Vitro

**FIGURE 4:**
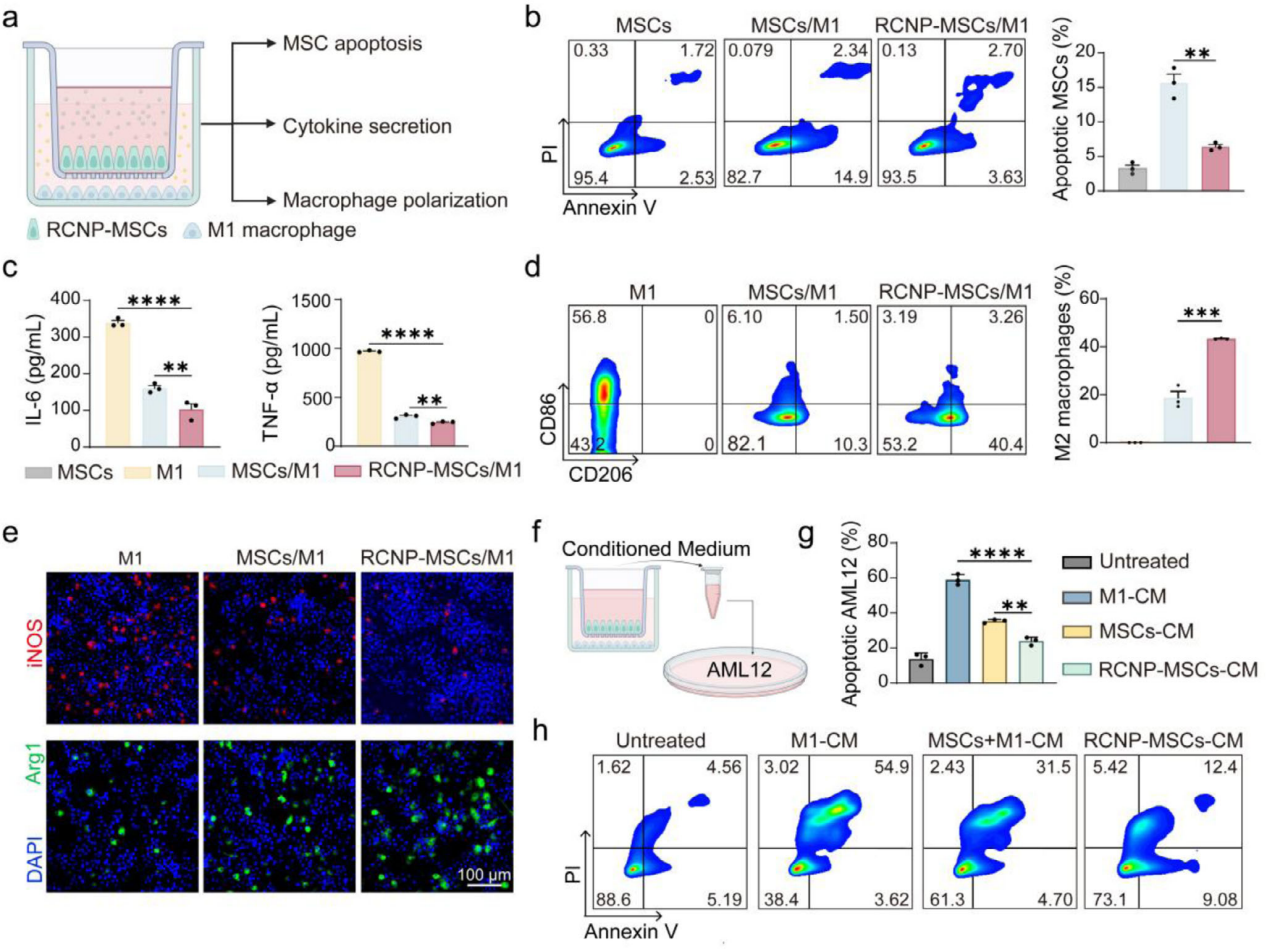
RCNP‐MSCs regulate macrophage polarization and protect hepatocytes. (a) Schematic representation of the experimental setup for evaluating the effects of RCNP‐MSCs on MSC apoptosis, cytokine secretion, and macrophage polarization using a transwell co‐culture system. (b) Flow cytometry analysis of apoptotic MSCs in different groups (MSCs, MSCs/M1, and RCNP‐MSCs/M1) using Annexin V/PI staining. “/” indicates coculture between the two indicated cell types. Means ± SEM, n = 3 independent experiments. (c) ELISA analysis of TNF‐α and IL‐6 levels in the co‐culture supernatants. Means ± SEM, n = 3 independent experiments. (d) Flow cytometric analysis of macrophage polarization (left) and quantification of the proportion of CD206^+^ M2 macrophages in each group (right). Means ± SEM, n = 3 independent experiments. (e) Representative immunofluorescence images of iNOS (red) and Arg1 (green) staining in macrophages. Nuclei were stained with DAPI (blue). (f) Schematic illustration of the experimental design for treating AML12 cells with conditioned medium (CM) collected from the co‐culture system. (g) Quantitative analysis of apoptosis in AML12 cells after treatment with conditioned medium from different groups. “CM” denotes conditioned medium collected from the corresponding culture system. MSC‐CM and RCNP‐MSCs‐CM denote conditioned media collected from M1 macrophages cocultured with MSCs or RCNP‐MSCs, respectively. Mean ± SEM, n = 3 independent experiments. (h) Representative flow cytometry plots of Annexin V/PI staining showing apoptosis in AML12 cells. For (b), (c), (d) and (g) statistical analysis was performed using one‐way ANOVA with Tukey's multiple‐comparisons test (^**^ *p* < 0.01; ^***^ *p* < 0.001; ^****^p < 0.0001). RCNP‐MSCs, MSCs modified with rosiglitazone‐loaded neutrophil‐mimetic nanoparticles; M1‐CM, conditioned medium from M1 macrophages; Native MSCs/M1‐CM, conditioned medium from the coculture of Native MSCs and M1 macrophages; RCNP‐MSCs/M1‐CM, conditioned medium from the coculture of RCNP‐MSCs and M1 macrophages.

Having established that rosiglitazone promotes MSC proliferation and enhances their therapeutic potential, and considering its intrinsic ability to modulate macrophage polarization [44], we next evaluated the immunoregulatory function of RCNP‐MSCs in vitro [45]. As shown in Figure 4a, a co‐culture system of M1 macrophages and RCNP‐MSCs was established to simultaneously assess MSC apoptosis, pro‐inflammatory cytokine secretion, and macrophage phenotypic changes. After 24 h of co‐culture, MSC apoptosis was first analyzed by flow cytometry. The results showed that co‐culture with M1 macrophages significantly induced MSC apoptosis, whereas the apoptotic rate of RCNP‐MSCs under the same conditions was markedly reduced (Figure 4b), indicating that RCNP surface modification effectively enhances MSC survival in an inflammatory microenvironment. Given that pro‐inflammatory cytokines released by M1 macrophages are key drivers of MSC apoptosis, we next measured the levels of these cytokines in the co‐culture supernatants. IL‐6 and TNF‐α levels were markedly elevated when M1 macrophages were cultured alone. Co‐culture with unmodified MSCs reduced the levels of both cytokines, whereas co‐culture with RCNP‐MSCs led to a further significant reduction (Figure 4c). These findings suggest that RCNP‐MSCs more effectively suppress the release of pro‐inflammatory cytokines under inflammatory conditions. As M1 macrophages are the main source of pro‐inflammatory cytokines [46], we next evaluated changes in macrophage phenotype within the co‐culture system. qPCR analysis showed that, coculture with Native MSCs increased the expression of the M2‐associated markers CD206 and Arg‐1 and decreased the expression of the M1‐associated markers TNF‐α and iNOS. These changes were more pronounced in the RCNP‐MSCs/M1 group (Figure S12a), indicating that RCNP‐MSCs more effectively promote macrophage polarization toward an anti‐inflammatory phenotype. To further explore the underlying mechanisms, we examined representative genes associated with PPARγ, NF‐κB, and STAT6/KLF4 signaling. Coculture with Native MSCs increased the expression of Cd36, Stat6, and Klf4 and decreased the expression of IL‐6 and IL‐1b, with more pronounced changes observed in the RCNP‐MSCs/M1 group (Figure S12b). These expression changes support the involvement of PPARγ‐, NF‐κB‐, and STAT6/KLF4‐related transcriptional programs in RCNP‐MSCs‐induced macrophage polarization. Flow cytometric quantification further confirmed that, compared with the unmodified MSC co‐culture group, the proportion of CD206^+^ M2 macrophages was significantly increased in the RCNP‐MSCs co‐culture group (Figure 4d), suggesting that RCNP‐MSCs promote macrophage polarization toward an anti‐inflammatory phenotype. Immunofluorescence staining further supported this conclusion, under RCNP‐MSCs co‐culture conditions, the number of iNOS^+^ M1 macrophages was significantly reduced, whereas the number of Arg1^+^ M2 macrophages was significantly increased (Figure 4e and Figure S13), further confirming the shift of macrophages toward an anti‐inflammatory phenotype.

To determine whether rosiglitazone protects MSCs directly or indirectly through modulation of macrophages, we performed additional experiments using M1 macrophage‐conditioned medium (M1‐CM) (Figure S14). For direct‐effect analysis, apoptosis was compared among Native MSCs + M1‐CM, Native MSCs + M1‐CM + free rosiglitazone (ROSI), and RCNP‐MSCs + M1‐CM. Free ROSI did not significantly reduce Native MSC apoptosis, and the apoptotic rate of RCNP‐MSCs was only slightly lower than that of Native MSCs under the same M1‐CM conditions, indicating that the direct protective effect of ROSI was relatively weak. For indirect‐effect analysis, apoptosis was compared among Native MSCs + M1‐CM, Native MSCs + ROSI‐M1‐CM, and Native MSCs + M1/RCNP‐MSCs‐CM. Specifically, ROSI‐M1‐CM was collected from ROSI‐treated M1 macrophages, whereas M1/RCNP‐MSCs‐CM was collected from M1 macrophages cocultured with RCNP‐MSCs. We found that both ROSI‐M1‐CM and M1/RCNP‐MSCs‐CM substantially reduced MSC apoptosis compared with M1‐CM, with M1/RCNP‐MSCs‐CM showing the strongest protective effect. Together, these results indicate that MSC protection was predominantly mediated by the indirect improvement of the macrophage‐derived inflammatory microenvironment.

To further determine whether the immunoregulatory effects of RCNP‐MSCs could be translated into functional protection of hepatocytes [47, 48], we collected conditioned medium from different co‐culture systems and used them to treat AML12 hepatocytes (Figure 4f). The results showed that M1‐CM markedly induced apoptosis in AML12 hepatocytes. In contrast, conditioned medium from MSCs co‐cultured with M1 macrophages (MSCs‐CM) alleviated hepatocyte apoptosis. Notably, conditioned medium from RCNP‐MSCs co‐cultured with M1 macrophages (RCNP‐MSCs‐CM) further reduced the apoptotic rate of AML12 cells (Figure 4g,h). Collectively, these results indicate that RCNP‐MSCs effectively modulate the immune microenvironment by reshaping macrophage phenotype and suppressing pro‐inflammatory cytokine release, thereby alleviating inflammation‐induced hepatocyte damage.

### 2.4 Homing and Hepatic Persistence of RCNP‐MSCs in ALF Mice

To improve the inflammatory targeting ability of MSCs, we coated rosiglitazone‐loaded PLGA nanoparticles with neutrophil membranes, taking advantage of the intrinsic inflammatory homing properties of neutrophil membranes [49]. Previous studies have shown that HL‐60 cells are a readily available cell source and can be differentiated into neutrophil‐like cells following DMSO treatment [34, 50]. Accordingly, DMSO‐differentiated HL‐60 cells were used as membrane donors. Flow cytometry showed that these cells were smaller, as indicated by reduced FSC, and expressed higher levels of CD11b (Figure S15a,b). Western blotting further revealed increased CD11b and CXCR2 levels in their membrane proteins, confirming the neutrophil‐like characteristics of the derived membranes (Figure S15c). We hypothesized that this neutrophil membrane‐based modification would enhance the chemotactic and adhesive behavior of MSCs toward inflammatory sites. To test this hypothesis, we first examined the adhesion of engineered MSCs to inflammation‐activated endothelial cells in vitro. HUVECs were pretreated with TNF‐α to mimic inflammatory endothelium and then incubated with MSCs [51]. Fluorescence microscopy showed that RCNP‐modified MSCs displayed significantly enhanced adhesion to activated HUVECs (Figure S16), supporting their potential for improved in vivo targeting.

**FIGURE 5:**
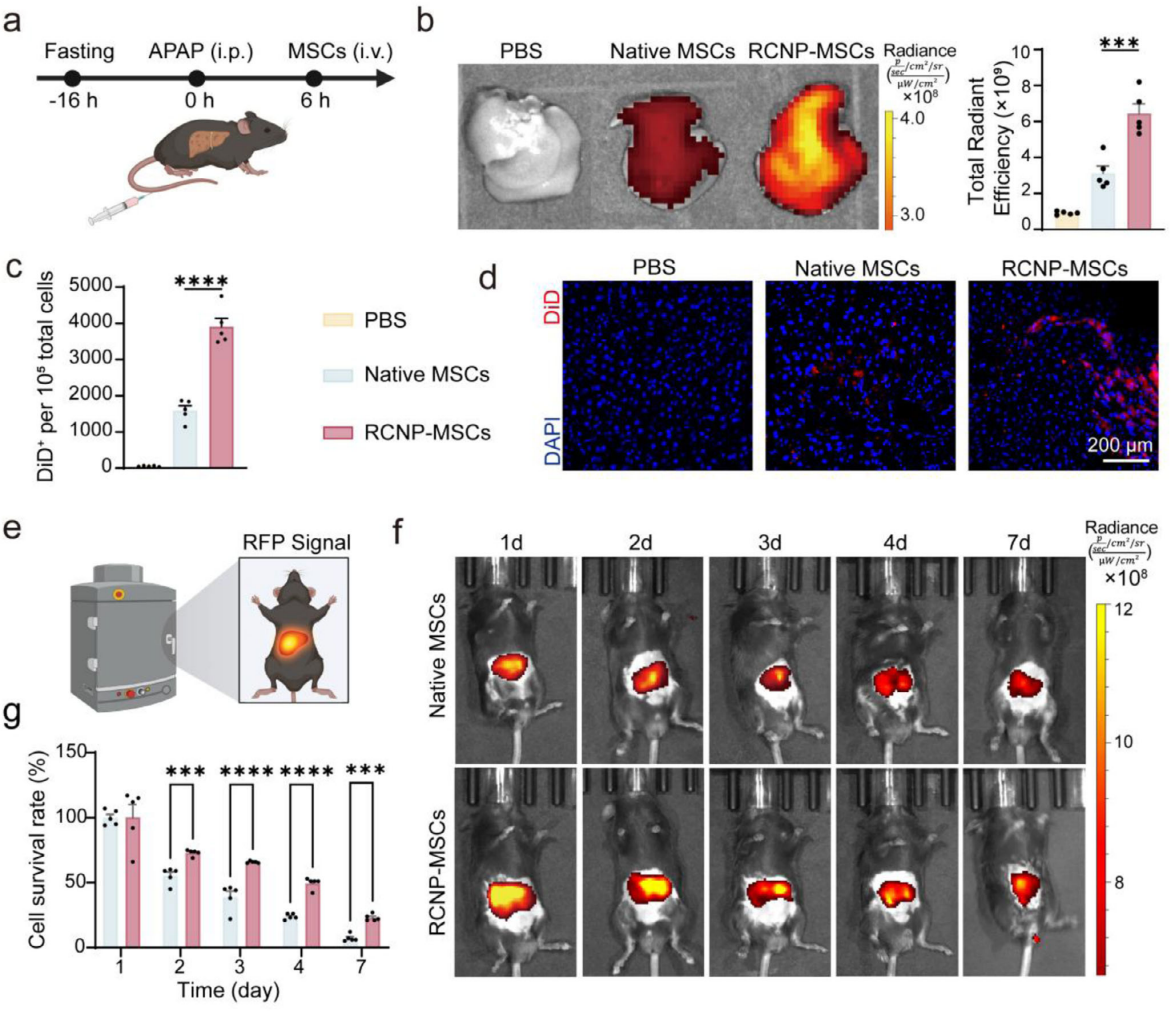
RCNP‐MSCs enhance accumulation and persistence in injured liver. (a) Schematic diagram of the experimental procedure showing that ALF was induced in mice by APAP injection, followed by intravenous injection of MSCs. (b) IVIS imaging of livers collected from ALF mice 24 h after treatment. Representative images show enhanced liver accumulation of RCNP‐MSCs, with quantification shown in the bar graph (right). Mice were euthanized 24 h after MSC injection, and livers were immediately harvested for imaging. Mean ± SEM, n = 5 mice. (c) Quantification of MSC homing to the liver. The bar graph shows the number of DiD^+^ cells among 1 × 10^5^ total cells isolated from ALF mouse livers 24 h after injection. Means ± SEM, n = 5 mice. (d) Representative immunofluorescence images of liver sections showing MSC homing in liver tissue. (e) Schematic illustration of in vivo fluorescence imaging of RFP‐labeled MSCs. (f) IVIS imaging of ALF mice treated with RFP‐labeled MSCs. Mice were anesthetized at 1, 2, 3, 4, and 7 days post‐transplantation and imaged using the IVIS system. (g) Quantification of fluorescence signal retention in the liver over time after transplantation. Means ± SEM, n = 5 mice. For (b), (c), statistical analysis was performed by one‐way ANOVA with Tukey's multiple‐comparisons test; for (g), statistical analysis was performed using Student's t‐test (^*^p < 0.05; ^**^ *p* < 0.01; ^****^ *p* < 0.0001). RCNP‐MSCs, MSCs modified with rosiglitazone‐loaded neutrophil‐mimetic nanoparticles.

Before conducting in vivo targeting experiments, we first assessed the biosafety of RCNP‐MSCs. Healthy mice were intravenously infused with RCNP‐MSCs, and after 14 days, heart, liver, spleen, lung, and kidney tissues were collected for histological evaluation. H&E staining showed no evident tissue damage in any of these organs (Figure S17), indicating favorable in vivo biosafety of RCNP‐MSCs. To determine whether RCNPs possess inherent inflammatory liver‐targeting capacity, DiD‐labeled PLGA, RPLGA, or RCNP were intravenously administered to acetaminophen (APAP)‐induced ALF mice. Ex vivo fluorescence imaging showed that RCNP exhibited greater accumulation in the injured liver than uncoated PLGA and RPLGA nanoparticles (Figure S18), demonstrating the inherent inflammatory liver‐targeting capacity of RCNP. We next evaluated the in vivo targeting performance of RCNP‐MSCs in an APAP‐induced ALF mouse model. 6 h after intraperitoneal APAP administration [52, 53, 54, 55], Vybrant‐DiD‐labeled MSCs were intravenously injected into the mice (Figure 5a). IVIS imaging performed 24 h after injection showed that RCNP‐MSCs accumulated to a greater extent in the injured liver than Native MSCs (Figure 5b). Flow cytometric quantification further revealed that the number of liver‐homed cells in the RCNP‐MSCs group was approximately 2.3‐fold that in the Native MSCs group (Figure 5c). Biodistribution analysis also showed that Native MSCs exhibited substantial nonspecific retention in the lungs, whereas RCNP‐MSCs displayed significantly reduced pulmonary retention and increased hepatic accumulation (Figure S19). Immunofluorescence staining of liver sections further demonstrated that RCNP‐MSCs migrated from the vascular lumen into the surrounding parenchymal tissue (Figure 5d). Collectively, these findings demonstrate that RCNP modification markedly improves the in vivo targeting and hepatic homing efficiency of MSCs in ALF mice.

To further evaluate the hepatic homing and in vivo persistence of MSCs, we used MSCs stably expressing red fluorescent protein (RFP) for dynamic tracking (Figure 5e). In vivo imaging revealed clear RFP fluorescence signals in the liver region after MSC injection, enabling longitudinal monitoring of MSC retention in the liver (Figure 5f). Notably, compared with Native MSCs, RCNP‐MSCs exhibited stronger and more sustained fluorescence signals in the liver at multiple time points. Quantitative analysis, using the liver fluorescence intensity on day 1 after injection as the baseline, showed that signal retention in the RCNP‐MSCs group on days 2, 3, 4, and 7 was significantly higher than that in the Native MSCs group (Figure 5g). These results indicate that RCNP engineering enhances the initial homing of MSCs to the injured liver while significantly prolonging their persistence and retention at the site of injury.

### 2.5 Evaluation of the In Vivo Therapeutic Efficacy of RCNP‐MSCs in ALF Mice

**FIGURE 6:**
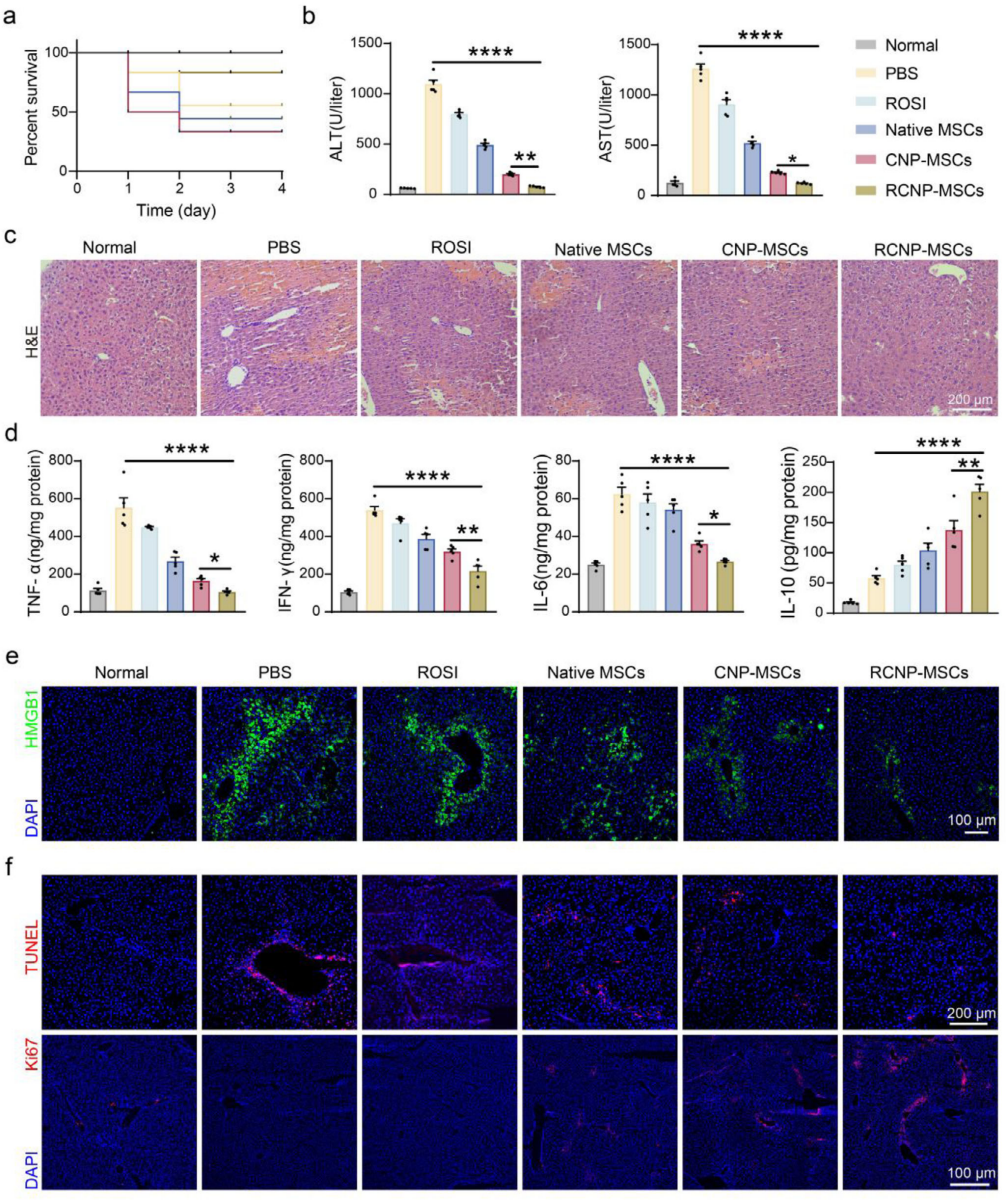
Therapeutic efficacy of RCNP‐MSCs in ALF mice. (a) Survival rates of ALF mice following various treatments. n = 10 mice. (b) Quantitative analysis of serum biochemical markers of liver injury on 4 days after treatment. Means ± SEM, n = 5 mice. (c) Representative H&E staining images of liver sections from ALF mice after different treatments. (d) Levels of TNF‐α, IFN‐γ, IL‐6, and IL‐10 in liver tissues after different treatments. Means ± SEM, n = 5 mice. (e) Representative immunofluorescence images of HMGB1 staining (green) in liver sections. (f) Representative TUNEL staining of apoptotic cells and Ki‐67 staining of proliferating cells in liver sections. For (b) and (d), statistical analysis was performed using one‐way ANOVA with Tukey's multiple‐comparisons test (^*^ *p* < 0.05; ^**^ *p* < 0.01; ^***^ *p* < 0.001; ^****^ *p* < 0.0001). ROSI, free rosiglitazone; CNP‐MSCs, MSCs modified with drug‐free neutrophil‐mimetic nanoparticles; RCNP‐MSCs, MSCs modified with rosiglitazone‐loaded neutrophil‐mimetic nanoparticles.

RCNP‐MSCs exhibited prolonged retention in the ALF mouse model, suggesting that the sustained release of rosiglitazone from the MSC surface may prolong the therapeutic activity of MSCs by improving the inflammatory microenvironment and reducing cell apoptosis. We therefore further evaluated the therapeutic efficacy of RCNP‐MSCs in the APAP‐induced ALF mouse model. To clarify the respective contributions of rosiglitazone and the nanoparticle carrier to the therapeutic outcome, free ROSI and MSCs modified with non‐drug‐loaded cell membrane‐coated nanoparticles (CNP‐MSCs) were included as controls. The free ROSI group was used to evaluate the therapeutic effect of rosiglitazone alone, whereas the CNP‐MSCs group allowed us to distinguish the contribution of rosiglitazone from that of the nanoparticle carrier. Given the rapid progression and high mortality of ALF, we first monitored mouse survival for 4 days. The results showed that the PBS group exhibited a mortality rate of 70%, whereas treatment with MSCs or RCNP‐MSCs significantly reduced mortality. Notably, the mortality rate in the RCNP‐MSCs group was only 10%, which was significantly lower than that in the other groups, indicating that this engineered modification markedly improved survival in ALF mice (Figure 6a). On day 4, serum alanine aminotransferase (ALT) and aspartate aminotransferase (AST) levels were measured to assess liver function. Compared with the PBS group, MSC treatment reduced both ALT and AST levels, confirming the protective effect of MSCs against APAP‐induced liver injury. Importantly, in the RCNP‐MSCs group, ALT and AST levels approached normal values, whereas these markers remained elevated in the other treatment groups, suggesting that RCNP‐MSCs were more effective in restoring liver function (Figure 6b). Gross examination of liver morphology further supported these findings. Compared with the healthy control group, the livers of PBS‐treated mice showed marked edema and pallor, whereas the livers of RCNP‐MSC‐treated mice displayed a more uniform dark brown color, indicating reduced tissue injury (Figure S20).

Since liver repair is typically accompanied by marked histological changes, we further evaluated tissue injury and regeneration by H&E staining of liver sections [56]. The results showed that the RCNP‐MSCs group exhibited the least structural damage, with markedly reduced necrotic areas and inflammatory infiltration, and an overall morphology more closely resembling that of the healthy control group (Figure 6c), indicating that RCNP‐MSCs effectively promote the repair of injured liver tissue. To further examine the regulatory effects of RCNP‐MSCs on the hepatic inflammatory microenvironment, we measured the levels of key inflammatory factors in liver tissue. ELISA results showed that RCNP‐MSCs treatment significantly reduced the levels of pro‐inflammatory cytokines, including TNF‐α, IFN‐γ, and IL‐6, while increasing the level of the anti‐inflammatory cytokine IL‐10 (Figure 6d), suggesting a pronounced anti‐inflammatory effect. As high‐mobility group box 1 (HMGB1) is a damage‐associated molecular pattern released from injured hepatocytes [57], we further evaluated hepatic HMGB1 by immunofluorescence staining. Representative immunofluorescence images showed that HMGB1 staining appeared weaker in the RCNP‐MSCs group than in the other treatment groups (Figure 6e), consistent with reduced hepatocellular injury. In addition, we assessed hepatocyte apoptosis and proliferation by TUNEL and Ki‐67 staining, respectively (Figure 6f). The number of TUNEL‐positive apoptotic cells was significantly decreased in the RCNP‐MSCs group, indicating a clear protective effect on hepatocytes. Moreover, Ki‐67 staining showed that treatment with RCNP‐MSCs significantly enhanced hepatocyte proliferation compared with the other groups, further supporting its role in promoting liver regeneration. Notably, across these therapeutic evaluations, free ROSI produced only partial improvement and was substantially less effective than RCNP‐MSCs (Figure 6), indicating that free rosiglitazone alone was insufficient to achieve the comprehensive therapeutic benefits observed with the cell–drug conjugate system.

In conclusion, these findings suggest that RCNP‐MSCs treatment markedly improves the survival of ALF mice and confers synergistic, multifaceted protective effects during liver repair, thereby exhibiting superior therapeutic efficacy compared with native MSCs.

### 2.6 Regulatory Effects of RCNP‐MSCs on Macrophage Phenotype In Vivo

**FIGURE 7:**
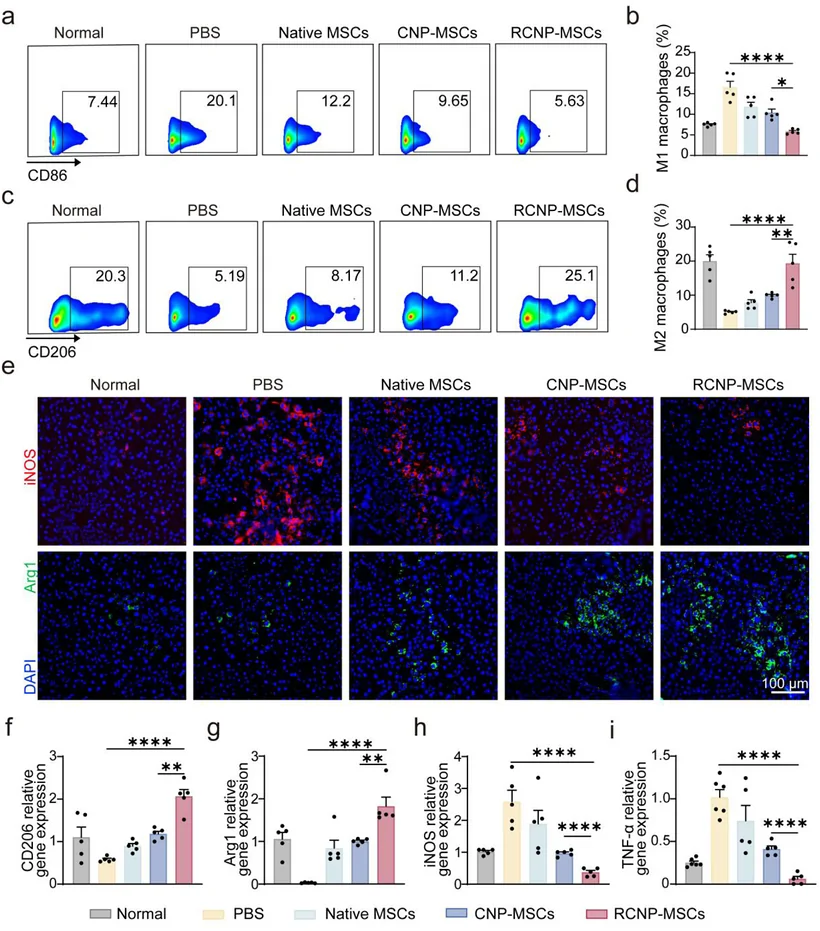
RCNP‐MSCs promote macrophage polarization toward an M2 phenotype in ALF mice. (a,b) Flow cytometric analysis (a) and quantification (b) of CD86^+^M1 macrophages within the total macrophage population. Means ± SEM, n = 5 mice. (c,d) Flow cytometric analysis (c) and quantification (d) of CD206^+^ M2 macrophages within the total macrophage population. Means ± SEM, n = 5 mice. (e) Representative immunofluorescence images of iNOS and Arg1 staining in liver sections. (f–i) qPCR analysis of the relative mRNA expression levels of CD206, Arg1, TNF‐α, and iNOS in liver tissues after different treatments. Means ± SEM, n = 5 mice. For (b), (d), and (f–i), statistical analysis was performed using one‐way ANOVA with Tukey's multiple‐comparisons test (^*^ *p* < 0.05; ^**^ *p* < 0.01; ^***^ *p* < 0.001; ^****^ *p* < 0.0001). Normal, healthy mice without ALF induction; Native MSCs, unmodified MSCs; CNP‐MSCs, MSCs modified with drug‐free neutrophil‐mimetic nanoparticles; RCNP‐MSCs, MSCs modified with rosiglitazone‐loaded neutrophil‐mimetic nanoparticles.

In ALF, activated M1 macrophages exacerbate the inflammatory response by secreting large amounts of pro‐inflammatory cytokines, thereby promoting hepatocyte injury and functional deterioration [58]. This process is a key driver of ALF progression. To assess the effect of RCNP‐MSCs on macrophage phenotype in the livers of ALF mice, we analyzed the proportions of pro‐inflammatory (M1) and anti‐inflammatory (M2) macrophages in different treatment groups. Flow cytometric analysis showed that, compared with the normal group, the PBS group exhibited a significantly increased proportion of CD86^+^ M1 macrophages in the liver. MSC treatment reduced the proportion of M1 macrophages, whereas RCNP‐MSCs treatment further decreased the proportion of CD86^+^ M1 macrophages (Figure 7a,b). In contrast, the proportion of CD206^+^ M2 macrophages was significantly reduced in the PBS group. MSC treatment partially restored the M2 macrophage population, while RCNP‐MSCs treatment significantly increased the proportion of CD206^+^ M2 macrophages to the highest level among all treatment groups (Figure 7c,d). These results indicate that RCNP‐MSCs more effectively suppress pro‐inflammatory macrophage activation and promote macrophage polarization toward an anti‐inflammatory phenotype.

To validate the flow cytometry findings at the tissue level, we performed immunofluorescence staining of liver sections. The results showed that the number of iNOS^+^ cells was markedly increased in the PBS group, whereas the number of Arg1^+^ cells was markedly reduced. In contrast, treatment with RCNP‐MSCs significantly decreased iNOS‐positive signals and increased Arg1‐positive signals, with spatial distribution patterns consistent with the flow cytometry results (Figure 7e), indicating that RCNP‐MSCs promote macrophage phenotypic transition toward an anti‐inflammatory phenotype in liver tissue. In addition, qPCR analysis of macrophage‐related marker genes in liver tissue showed that RCNP‐MSCs treatment significantly upregulated the expression of M2‐associated genes (CD206 and Arg1) and downregulated the expression of M1‐associated genes (iNOS and TNF‐α) (Figure 7f–i), further confirming its regulatory effect on macrophage phenotype at the transcriptional level.

Collectively, these results demonstrate that RCNP‐MSCs treatment effectively inhibits pro‐inflammatory macrophage activation in the livers of ALF mice while promoting macrophage polarization toward an anti‐inflammatory phenotype, thereby contributing to the improvement of the hepatic inflammatory microenvironment.

## 3 Conclusion

To address the low survival and limited therapeutic efficacy of MSCs in the treatment of ALF, we designed and constructed a cell–drug conjugate, RCNP‐MSCs, using MSCs as delivery vehicles. This strategy enabled the synergistic integration of rosiglitazone delivery with cell therapy. Mechanistically, rosiglitazone activated the PI3K–Akt signaling pathway, thereby enhancing MSC proliferation and optimizing their paracrine profile. In vivo imaging demonstrated that RCNP‐MSCs exhibited greater targeted accumulation and more sustained retention in the injured liver. In an APAP‐induced ALF mouse model, RCNP‐MSCs treatment significantly improved survival, alleviated liver tissue injury, and exerted hepatoprotective effects by inhibiting hepatocyte apoptosis and promoting hepatocyte regeneration. Further analyses revealed that RCNP‐MSCs regulated hepatic macrophage phenotype by promoting polarization from the pro‐inflammatory M1 phenotype toward the anti‐inflammatory M2 phenotype, thereby improving the local immune microenvironment in the liver. Collectively, these findings demonstrate that the cell–drug conjugate strategy proposed in this study markedly enhances the therapeutic efficacy of MSCs in ALF and provides a scalable approach for the synergistic application of small‐molecule drugs and cell therapies.

## 4 Experimental Section

### 4.1 Ethical Statement

All animal experiments were conducted in strict accordance with the Guide for the Care and Use of Laboratory Animals. All procedures were approved by the Animal Care and Use Committee of South China University of Technology (Approval No. 2022020).

### 4.2 Animals

Male C57BL/6 mice (6–8 weeks old, weighing 20–25 g) were purchased from Hunan SJA Laboratory Animal Co., Ltd. (Hunan, China) and housed in designated specific pathogen‐free (SPF) animal facilities. All mice were maintained under controlled conditions with a 12‐hour/12‐hour light/dark cycle, a temperature of 22°C, and humidity levels ranging from 45% to 60%. Mice were allowed free access to food and water.

### 4.3 Materials

Acetaminophen (APAP; Cat# A105809) and Percoll density gradient medium (Cat# P331004) were purchased from Aladdin (Shanghai, China). Rosiglitazone (Cat# 01153223) was obtained from Shanghai Tansuo Biotechnology Co., Ltd. (Shanghai, China). Lipopolysaccharides (LPS; Cat# L2630) were purchased from Sigma–Aldrich (St. Louis, MO, USA). DSPE‐PEG_2000_‐DBCO (Cat# LZ‐DS‐GN0045) was procured from Chongqing Yusi Pharmaceutical Technology Co., Ltd. (Chongqing, China). PLGA (50:50, 0.67 dL/g; Lactel Absorbable Polymers, USA) was purchased through Xi'an Qiyue Biotechnology Co., Ltd. (Xi'an, China). 1,3,4,6‐Tetra‐O‐acetyl‐N‐azidoacetyl‐D‐mannosamine (Ac_4_ManNAz; CAS: 361154‐30‐5) was obtained from Click Chemistry Tools (Scottsdale, AZ, USA). LY294002 (Cat# HY‐10108) was purchased from MedChemExpress (Monmouth Junction, NJ, USA). Dulbecco's Modified Eagle's Medium (DMEM; high glucose and low glucose), RPMI 1640, trypsin‐EDTA, and penicillin‐streptomycin were procured from Invitrogen (Thermo Fisher Scientific, Waltham, MA, USA). Fetal bovine serum (FBS) was obtained from Biological Industries (Beit Haemek, Israel). The following kits were purchased from Beyotime Biotechnology (Shanghai, China): Cell Counting Kit‐8 (CCK‐8, Cat# C0037), Hoechst 33342 live‐cell staining solution (Cat# C1028), DiO cell‐labeling solution (Cat# C1933), Annexin V‐FITC Apoptosis Detection Kit (Cat# C1062), Ki‐67 Cell Proliferation Assay Kit (Cat# C2312), Enhanced BCA Protein Assay Kit (Cat# P0010), and Calcein/PI Cell Viability/Cytotoxicity Assay Kit (Cat# C2015). The lipophilic fluorescent dye DiD (Cat# D307) was obtained from Invitrogen (Thermo Fisher Scientific, Waltham, MA, USA). The One‐step TUNEL In Situ Apoptosis Kit (Cat# E‐CK‐A322) was procured from Elabscience (Wuhan, China). Hematoxylin and eosin (H&E) staining solution (Cat# G1005) was obtained from Servicebio (Wuhan, China). ELISA kits were purchased from Shanghai Enzyme‐linked Biotechnology Co., Ltd. (Shanghai, China). The RNAeasy Animal RNA Extraction Kit (Cat# M5105) was obtained from Sinomab Biotechnology Co., Ltd. (Beijing, China). HiFiScript gDNA Removal RT MasterMix (Cat# CW2020M) and MagicSYBR Mixture (Cat# CW3008M) were obtained from CoWin Biosciences Co., Ltd. (Beijing, China). The anti‐iNOS antibody (Cat# ab3523) was purchased from Abcam (Cambridge, UK), and the anti‐Arg1 antibody (Cat# 93668) was obtained from Cell Signaling Technology (Danvers, MA, USA). Flow cytometry antibodies (Table S1) were purchased from BioLegend (San Diego, CA, USA). Oligonucleotide primers were synthesized and purified by General Biol Co., Ltd. (Chuzhou, China), and their specific sequences are listed in Table S2.

### 4.4 Cell Culture

HUVECs [American Type Culture Collection (ATCC), PCS‐100‐013] were cultured in high‐glucose DMEM containing 10% FBS, supplemented with 1% penicillin and 1% streptomycin. HL‐60 cells (ATCC, CCL‐240) were cultured in RPMI 1640 medium containing 10% FBS, supplemented with 1% penicillin and 1% streptomycin. RAW264.7 cells (STCC20020P‐1, Cellverse Bioscience Technology, Shanghai, China) were cultured in high‐glucose DMEM containing 10% FBS, supplemented with 1% penicillin and 1% streptomycin. M1 macrophages were induced by stimulation with lipopolysaccharide (LPS, 1 µg/mL; Sigma–Aldrich, USA) for 24 h. AML12 cells (iCell‐m003, iCell Bioscience Inc., Shanghai, China) were cultured in DMEM/F‐12 medium supplemented with 1% insulin‐transferrin‐selenium (ITS), dexamethasone (40 ng/mL), and 1% GlutaMAX. Mouse bone marrow‐derived mesenchymal stromal cells (shanghai Jinyuan Biotechnology Co., Ltd, Cat# JY‐J631) were cultured in low‐glucose DMEM containing 10% FBS, supplemented with 1% penicillin and 1% streptomycin. All cells were maintained at 37°C in a 5% CO_2_ incubator with 95% relative humidity. Experiments were conducted using MSCs from passages 3 to 6.

### 4.5 Neutrophil Cell Membrane Derivation

HL‐60 cells were washed with phosphate‐buffered saline (PBS) three times (centrifugation at 800 g for 5 min at 4°C). The cells were resuspended in a hypotonic lysing buffer containing 30 mM Tris‐HCl (pH 7.5), 225 mM D‐mannitol, 75 mM sucrose, 0.2 mM EGTA (Sigma–Aldrich, St. Louis, MO, USA), and a protease and phosphatase inhibitor cocktail (Thermo Fisher Scientific, Waltham, MA, USA). Cells were disrupted using a Dounce homogenizer with a tight‐fitting pestle (20 passes).The homogenate was centrifuged at 20 000 g for 25 min at 4°C. The pellet was discarded, and the supernatant was ultracentrifuged at 100 000 g for 60 min at 4°C. The resulting pellet, containing the cell membrane fraction, was washed twice with deionized water containing 0.2 mM EDTA. Membrane protein content was quantified using a BCA Protein Assay Kit with bovine serum albumin (BSA) as the standard. The purified membranes were suspended in 0.2 mM EDTA to a protein concentration of 4 mg/mL and stored at −80°C for subsequent studies.

### 4.6 Synthesis of Rosiglitazone‐Loaded Neutrophil‐Mimetic Nanoparticles (RCNP)

Rosiglitazone‐loaded neutrophil‐mimetic nanoparticles (RCNP) were synthesized via a nanoprecipitation method followed by membrane coating. Briefly, PLGA was dissolved in acetone at a concentration of 20 mg/mL, and rosiglitazone was added to the PLGA organic phase at a drug‐to‐polymer weight ratio of 1:4. A total volume of 0.5 mL of the PLGA/rosiglitazone solution was added dropwise into 5 mL of deionized water under continuous stirring. The organic solvent was then removed under vacuum to allow complete evaporation of acetone, yielding rosiglitazone‐loaded PLGA nanoparticle cores (RPLGA). For membrane coating, the purified HL‐60 cell membranes were mixed with RPLGA at a polymer‐to‐membrane protein weight ratio of 2:1. The mixture was sonicated using a bath sonicator (Fisher Scientific FS30D, Waltham, MA, USA) for 2 min. The resulting rosiglitazone‐loaded neutrophil‐mimetic nanoparticles (RCNP) were purified by centrifugation (11 000 g, 20 min), washed with 1× PBS to remove excess membranes, and finally resuspended in 1× PBS for further use. Blank nanoparticles (CNPs) were synthesized using the same procedure without the addition of rosiglitazone.

### 4.7 Characterization of RCNP

The hydrodynamic diameter, polydispersity index (PDI), and zeta potential of RCNP were determined using dynamic light scattering (DLS) with a Zetasizer Nano ZS (Malvern Instruments, Malvern, UK). Measurements were performed in triplicate at 25°C. For morphological observation, nanoparticle samples were deposited onto carbon‐coated copper grids (200 mesh) and negatively stained with 0.2% (w/v) uranyl acetate for 1 min. The grids were air‐dried and visualized using a transmission electron microscope (Talos F200C, Thermo Fisher Scientific, Waltham, MA, USA) operated at an accelerating voltage of 200 kV.

### 4.8 Surface Engineering of MSCs

DSPE‐PEG_2000_‐DBCO was dissolved in deionized water and added to the RCNP suspension at a weight ratio of 0.5 (DSPE‐PEG_2000_‐DBCO/PLGA). The mixture was gently stirred for 2 h in an ice bath to facilitate the insertion of DSPE‐PEG_2000_‐DBCO into the nanoparticle membrane. Subsequently, the suspension was centrifuged at 11 500 g for 20 min at 4°C to remove excess DSPE‐PEG_2000_‐DBCO. The purified DBCO‐functionalized nanoparticles were collected from the pellet and denoted as DBCO‐RCNP. MSCs were cultured to 40%–50% confluence, after which the culture medium was replaced with fresh medium supplemented with 40 µM Ac_4_ManNAz. Cells were continuously incubated under standard culture conditions (37°C, 5% CO_2_) for 72 h to generate azide‐modified MSCs (N_3_‐MSCs). DBCO‐RCNP were resuspended in serum‐free culture medium at a final concentration of 1 mg/mL (total nanoparticle mass concentration). 1×10^7^ N_3_‐MSCs were washed with 1× PBS and incubated with DBCO‐RCNP (1 mL per well) for approximately 3 h to allow bioorthogonal conjugation between azide and DBCO groups. Following incubation, cells were washed three times with 1× PBS to remove unbound nanoparticles, yielding the engineered MSCs, hereafter referred to as RCNP‐MSCs.

### 4.9 Evaluation of Surface Stability on RCNP‐MSCs

MSCs were incubated with DiO‐labeled RCNP to allow surface modification. After incubation, cells were washed three times with 1× PBS to remove unbound nanoparticles and subsequently cultured in complete medium for 0, 24, and 48 h. At each time point, nuclei were counterstained with Hoechst 33342. Fluorescence imaging was performed using a confocal laser scanning microscope (LSM 880 with Airyscan, Carl Zeiss, Oberkochen, Germany). The mean fluorescence intensity per cell was quantified at 0, 24, and 48 h using ZEN software (Carl Zeiss). For flow cytometric analysis, RCNP‐MSCs were harvested at 0, 5, 12, 24, and 48 h, washed, and resuspended in 1× PBS. For retention analysis, the mean fluorescence intensity (MFI) of the cells was determined. For fluorescence quenching analysis, cells from parallel samples at each time point were incubated with trypan blue solution (0.25 mg/mL in HBSS, 100 µL) for 1 min to quench extracellular fluorescence. After washing three times with 1× PBS to remove the quenching solution, cells were analyzed immediately using a BD LSRFortessa flow cytometer (BD Biosciences, San Jose, CA, USA). Data were analyzed using FlowJo software.

### 4.10 Quantitative Determination of RCNP Conjugation Density on MSCs Surface

The particle concentration and protein content of the same batch of RCNP were determined by nanoparticle tracking analysis (NTA) and the BCA assay, respectively, to establish the relationship between RCNP particle number and protein content. After RCNP conjugation, the incubation supernatant and wash fractions were collected, and the amount of unbound RCNP was measured by the BCA assay. The amount of cell‐associated RCNP was determined by subtracting the unbound amount from the initial input and was then converted to particle number based on the established relationship. The resulting particle number was divided by the total number of MSCs to calculate the average number of RCNP particles conjugated per cell.

### 4.11 Cell Viability Assay (CCK‐8)

MSCs were seeded into 96‐well plates at a density of 3,000 cells per well and cultured overnight to allow for cell adhesion. The culture medium was then replaced with fresh medium containing rosiglitazone (0.1–2 µM) at the indicated concentrations. After 24 h of treatment, cell viability was assessed using the Cell Counting Kit‐8 according to the manufacturer's instructions.

### 4.12 In Vitro Co‐Culture Model and Macrophage Polarization

To simulate an inflammatory microenvironment in vitro, a Transwell‐based co‐culture system was established using Raw264.7 cells and MSCs. Briefly, Raw264.7 cells were seeded into the lower chambers of 24‐well plates at a density of 5 × 10^4^ cells per well and cultured at 37°C in a humidified atmosphere containing 5% CO_2_ for 24 h. To induce inflammatory activation, Raw264.7 cells were stimulated with lipopolysaccharide (LPS, 1 µg/mL) for an additional 24 h. Subsequently, native MSCs or engineered MSCs (5 × 10^4^ cells per well) were seeded into the upper chambers of Transwell inserts (0.8 µm pore size; Corning) and co‐cultured with LPS‐activated Raw264.7 cells for 24 h. Prior to co‐culture, the LPS‐containing medium in the lower chambers was removed, and the cells were washed with 1× PBS to eliminate residual LPS. After co‐culture, MSCs in the upper chambers were harvested, and apoptosis was evaluated using an Annexin V–FITC Apoptosis Detection Kit according to the manufacturer's instructions. Samples were analyzed by flow cytometry. Raw264.7 cells in the lower chambers were collected for polarization analysis. For flow cytometry, cells were stained with fluorophore‐conjugated antibodies against CD86 and CD206. For immunofluorescence staining, Raw264.7 cells were fixed, permeabilized, and incubated with primary antibodies against inducible nitric oxide synthase (iNOS) and arginase‐1 (Arg1), followed by incubation with appropriate fluorophore‐conjugated secondary antibodies.

### 4.13 In Vitro Hepatocyte Protection Assay

To further evaluate the hepatoprotective efficacy of engineered MSCs under inflammatory conditions, a conditioned medium (CM)‐based assay was performed. Briefly, culture supernatants from the co‐culture system described above were collected after 24 h. The supernatants were centrifuged at 3,000 g for 5 min to remove cellular debris and used as conditioned medium (CM). AML12 hepatocytes were seeded at a density of 5 × 10^4^ cells per well in DMEM/F‐12 medium. After 12 h of incubation to allow cell attachment, the culture medium was replaced with the collected CM, and cells were incubated for an additional 24 h. Subsequently, hepatocyte apoptosis was evaluated using an Annexin V–FITC Apoptosis Detection Kit according to the manufacturer's instructions. Samples were analyzed by flow cytometry to quantify apoptosis rates.

### 4.14 In Vivo Safety Evaluation

Healthy male C57BL/6 mice were randomly divided into five groups: Normal, PBS, Native MSCs, CNP‐MSCs, and RCNP‐MSCs (n = 3 mice per group). Mice in the three cell‐treatment groups received 1 × 10^6^ corresponding MSCs via tail‐vein injection, whereas the PBS group received an equal volume of PBS and the Normal group received no treatment. Fourteen days after administration, the mice were euthanized, and the heart, liver, spleen, lung, and kidney were collected, fixed in 4% paraformaldehyde, embedded in paraffin, sectioned, and stained with H&E. Histological changes in the major organs were examined to evaluate in vivo safety.

### 4.15 Establishment of APAP‐Induced ALF Model

Acetaminophen was dissolved in sterile physiological saline at 50°C to prepare a stock solution. Male C57BL/6 mice (6–8 weeks old, 22–24 g) were fasted for 16 h prior to the experiment with free access to water. Acute liver failure was induced by a single intraperitoneal (i.p.) injection of APAP at a dose of 500 mg/kg. Mice were allowed to resume a normal diet immediately after injection. The general health status and survival of mice were monitored continuously. At designated time points, peripheral blood was collected via retro‐orbital puncture and centrifuged to obtain serum. Serum levels of ALT and AST were measured using an automatic biochemical analyzer (Hitachi, Tokyo, Japan). Mouse organs were harvested and subjected to hematoxylin and eosin (H&E) staining.

### 4.16 IVIS Imaging of the Mouse Liver

Six hours after the establishment of the ALF model, RFP‐labeled engineered MSCs (1 × 10^6^ cells) were administered via the tail vein. Prior to imaging, abdominal hair was removed to minimize background autofluorescence. At 1, 2, 3, 4, and 7 days post‐injection, mice were anesthetized with isoflurane and imaged using an IVIS Lumina Series III in vivo imaging system (PerkinElmer, Waltham, MA, USA) to monitor the in vivo distribution and retention of transplanted MSCs.

### 4.17 Biodistribution Analysis

To evaluate the tissue distribution of engineered MSCs in vivo, mice were randomly divided into three groups after ALF induction and intravenously injected with 1× PBS, 1 × 10^6^ native MSCs, or 1 × 10^6^ engineered MSCs. For cell tracking, native MSCs and engineered MSCs were labeled with the lipophilic fluorescent dye DiD according to the manufacturer's instructions prior to administration. At 24 h post‐injection, mice were sacrificed, and major organs, including the heart, liver, spleen, lung, and kidney, were harvested for ex vivo fluorescence imaging. Imaging was performed using an IVIS Lumina Series III in vivo imaging system (PerkinElmer, Waltham, MA, USA), and fluorescence intensity was quantified using Living Image software (v4.4, PerkinElmer).

### 4.18 Preparation of Liver Single‐Cell Suspensions

Liver tissue was enzymatically digested to obtain single cells. Briefly, the tissue was minced and resuspended in RPMI‐1640 supplemented with collagenase IV (1 mg/mL) and DNase I (0.1 mg/mL). After incubation on a shaker at 120 rpm for 15 min, the suspension was filtered through a 200‐mesh nylon mesh to remove undigested tissue fragments. The cells were then collected by centrifugation at 300 × g for 5 min at 4°C. Following cell counting, the cells were stained with antibodies.

### 4.19 Enzyme‐Linked Immunosorbent Assay (ELISA)

To evaluate the paracrine profile of MSCs, cells were washed with PBS, and the culture medium was replaced with serum‐free medium to eliminate interference from bovine serum proteins. After 72 h of incubation under serum‐free conditions, culture supernatants were collected and centrifuged at 3,000 g for 15 min at 4°C to remove cellular debris. The levels of secreted paracrine factors, including FGF‐2, HGF, SDF‐1, VEGF, MCP‐1, and IL‐10, were then quantified using commercial ELISA kits (Shanghai Enzyme‐linked Biotechnology Co., Ltd., Shanghai, China) according to the manufacturer's instructions. For liver tissue analysis, liver samples were weighed and homogenized in cold 1× PBS containing a protease inhibitor cocktail to prevent protein degradation. The homogenates were centrifuged at 10 000 g for 15 min at 4°C, and the supernatants were collected. Inflammatory cytokine levels, including TNF‐α, IFN‐γ, IL‐6, and IL‐10, were quantified using ELISA kits. Cytokine levels in liver tissues were normalized to total protein content, which was determined using a BCA Protein Assay Kit (Shanghai Enzyme‐linked Biotechnology Co., Ltd.).

### 4.20 Flow Cytometry to Detect Hepatic Macrophages in Liver Tissue

After mice were euthanized by exsanguination, livers were harvested and weighed. Liver tissues were gently dissociated through a 200‐mesh stainless steel strainer in cold 1× PBS to obtain single‐cell suspensions. The cell suspensions were transferred to 15 mL centrifuge tubes and centrifuged at 450 g for 5 min. After removal of the supernatant, cell pellets were resuspended in 8 mL of 40% Percoll solution and centrifuged at 800 g for 20 min for density gradient separation. Following centrifugation, the upper Percoll layer containing cellular debris was discarded, and the cell pellets were transferred to new 15 mL tubes. Red blood cells were lysed by incubation with 2 mL of red blood cell lysis buffer at 4°C for 5 min, and the reaction was terminated by adding 10 mL of 1× PBS. Cells were then collected by centrifugation at 450 g for 5 min, resuspended in 1× PBS, and counted. Prior to staining, cells were incubated with anti‐mouse CD16/32 (Fc block) for 10 min on ice to reduce non‐specific binding. Finally, cells were stained with appropriate fluorophore‐conjugated antibodies and analyzed by flow cytometry to quantify hepatic macrophages.

### 4.21 Statistical Analysis

Statistical analyses were performed using GraphPad Prism version 10.1.2. Unless otherwise stated, quantitative data are presented as the mean ± standard error of the mean (SEM). The exact sample size (n) and whether n represents independent experiments, biological samples, or animals are specified in the corresponding figure legends. No data transformation was performed unless otherwise indicated. Comparisons between two independent groups were performed using an unpaired two‐tailed Student's t‐test. Comparisons among three or more groups were performed using ordinary one‐way analysis of variance (ANOVA), followed by Tukey's multiple‐comparisons test. Statistical significance was defined as p < 0.05, with significance indicated as follows: ^*^ *p* < 0.05, ^**^ *p* < 0.01, ^***^ *p* < 0.001, and ^****^ *p* < 0.0001; ns, not significant.

## Author Contributions

T.Y. designed and performed the experiments, analyzed the data, and wrote the manuscript. J.W. performed the experiments, interpreted the data, and edited the manuscript. Z.W., X.L., and L.Y. performed animal studies. J.L. performed the experiments. P.S. conceived the concept, designed the study, interpreted the data, and wrote the manuscript.

## Conflicts of Interest

The authors declare no conflicts of interest.

## Supporting information




**Supporting File**: advs77411‐sup‐0001‐SuppMat.docx.

## Data Availability

The data that support the findings of this study are available from the corresponding author upon reasonable request.
