# Supplementary material for: Mesenchymal Stromal Cell‐Based Cell–Drug Conjugates for the Treatment of Acute Liver Failure
Source: Adv Sci (Weinh). 2026 Aug 25:e77411. Online ahead of print. doi: 10.1002/advs.77411 (PMC13505584; doi:10.1002/advs.77411)
Supplement: Supplementary file 1 [file ADVS-9999-e77411-s001.docx]

**Supporting Information**

**Mesenchymal stromal cell-based cell–drug conjugates for the treatment of acute liver failure**

**Supplementary Tables**

Supplementary Table 1. Antibodies used for flow cytometry.

Supplementary Table 2. Primer sequences.

**Supplementary Figures**
Supplementary Figure 1. Polydispersity indices of RPLGA, Membrane, and RCNP.

Supplementary Figure 2. Characterization of drug loading and encapsulation efficiency.

Supplementary Figure 3. In vitro drug release kinetics.

Supplementary Figure 4. Optimization of RCNP loading on MSCs. Quantification of RCNP binding.

Supplementary Figure 5. Visual confirmation of RCNP surface modification on MSCs.

Supplementary Figure 6. Stability of RCNP anchored on the MSC surface.

Supplementary Figure 7. Effects of surface engineering on MSC biological functions.

Supplementary Figure 8. Gene expression of PPARγ and Cyclin D1 in ROSI-treated MSCs.

Supplementary Figure 9. PCA and KEGG pathway enrichment analysis of ROSI-treated MSCs.

Supplementary Figure 10. Cell viability of MSCs treated with different concentrations of rosiglitazone.

Supplementary Figure 11. Effect of rosiglitazone treatment on HGF and FGF-2 secretion.

Supplementary Figure 12. RCNP-MSCs regulate macrophage polarization and associated transcriptional programs.

Supplementary Figure 13. Quantitative immunofluorescence analysis of macrophage polarization markers.

Supplementary Figure 14. Direct and indirect effects of rosiglitazone on MSC apoptosis under M1 macrophage-conditioned inflammatory conditions.

Supplementary Figure 15. Characterization of DMSO-differentiated HL-60 cells and neutrophil-associated membrane proteins used for nanoparticle coating.

Supplementary Figure 16. Representative fluorescence images and quantitative analysis showing the adhesion of MSCs to HUVECs.

Supplementary Figure 17. Toxicity analysis.

Supplementary Figure 18. Hepatic accumulation of free RCNP in APAP-induced acute liver failure mice.

Supplementary Figure 19. Biodistribution of MSCs in ALF mice.

Supplementary Figure 20. Macroscopic liver appearance.

| Product | Catalogue Number | Supplier |
| --- | --- | --- |
| anti-mouse CD16/32 | 101301 | Biolegend |
| FITC anti-mouse CD45.2 | 109806 | Biolegend |
| APC anti-mouse F4/80 | 123116 | Biolegend |
| PE anti-mouse CD86 | 105007 | Biolegend |
| PE/Cyanine7 anti-mouse CD206 | 141719 | Biolegend |

Supplementary Table 1. Antibodies used for flow cytometry.

| Genes |  | Sequence (5’-3’) |
| --- | --- | --- |
| Akt1 | Forward | ATCCCCTCAACAACTTCTCAGT |
| Akt1 | Reverse | CTTCCGTCCACTCTTCTCTTTC |
| Arg-1 | Forward | CTCCAAGCCAAAGTCCTTAGAG |
| Arg-1 | Reverse | AGGAGCTGTCATTAGGGACATC |
| Bcl-2 | Forward | CTGGCATCTTCTCCTTCCAG |
| Bcl-2 | Reverse | GACGGTAGCGACGAGAGAAG |
| CD206 | Forward | CTCTGTTCAGCTATTGGACGC |
| CD206 | Reverse | CGGAATTTCTGGGATTCAGCTTC |
| Cyclin D1 | Forward | GAGGTTCCTGTTCACAATACCTCA |
| Cyclin D1 | Reverse | AGACCGCCCACCTGCC |
| Gapdh | Forward | AGGTCGGTGTGAACGGATTTG |
| Gapdh | Reverse | TGTAGACCATGTAGTTGAGGTCA |
| HO-1 | Forward | GGTCAGGTGTCCAGAGAA |
| HO-1 | Reverse | CTTCCAGGGCCGTGTAGATA |
| Cd36 | Forward | TCCTCTGACATTTGCAGGTCTATC |
| Cd36 | Reverse | AAAGGCATTGGCTGGAAGAA |
| IL-1b | Forward | GCAACTGTTCCTGAACTCAACT |
| IL-1b | Reverse | ATCTTTTGGGGTCCGTCAACT |
| Stat6 | Forward | TCTCCACGAGCTTCACATTG |
| Stat6 | Reverse | GACCACCAAGGGCAGAGAC |
| Klf4 | Forward | GGTGCAGCTTGCAGCAGTAA |
| Klf4 | Reverse | AAAGTCTAGGTCCAGGAGGTCGTT |
| IL-10 | Forward | GCTCTTACTGACTGGCATGAG |
| IL-10 | Reverse | CGCAGCTCTAGGAGCATGTG |
| IL-6 | Forward | TAGTCCTTCCTACCCCAATTTCC |
| IL-6 | Reverse | TTGGTCCTTAGCCACTCCTTC |
| Nos2 | Forward | GTTCTCAGCCCAACAATACAAGA |
| Nos2 | Reverse | GTGGACGGGTCGATGTCAC |
| Ppia | Forward | GAGCTGTTTGCAGACAAAGTTC |
| Ppia | Reverse | CCCTGGCACATGAATCCTGG |
| TGFβ | Forward | ATCCTCAAGTTGCACCCTTATCT |
| TGFβ | Reverse | AAAGAGCCTTCGGTGGATTGC |
| TNF-α | Forward | ACGGCATGGATCTCAAAGAC |
| TNF-α | Reverse | AGATAGCAAATCGGCTGACG |
| TSG6 | Forward | GGCTGGCAGATACAAGCTCA |
| TSG6 | Reverse | TCAAATTCACATACGGCCTTGG |

Supplementary Table 2. Primer sequences.

Supplementary Figure 1. Polydispersity indices of RPLGA, Membrane, and RCNP. The PDI values were determined by dynamic light scattering. Mean ± SEM, n = 3 independent experiments.

Supplementary Figure 2. Characterization of drug loading and encapsulation efficiency. a) Encapsulation efficiency of RPLGA and RCNP. b) Drug loading capacity of RPLGA and RCNP. Means ± SEM, n = 3 independent experiments. Statistical analysis was performed using Student's t-test (n.s., not significant).

Supplementary Figure 3. In vitro drug release kinetics. In vitro release kinetics of rosiglitazone from RPLGA and RCNP in PBS (pH = 7.4) over 60 hours. Means ± SEM, n = 3 independent experiments. Statistical analysis was performed using Student's t-test (**P* < 0.05).

Supplementary Figure 4. Optimization of RCNP loading on MSCs. Quantification of RCNP binding. a) and rosiglitazone loading b) on 1×10^7^ MSCs at various initial RCNP inputs. Means ± SEM, n =3 independent experiments.

Supplementary Figure 5. Visual confirmation of RCNP surface modification on MSCs. Representative brightfield (BF) and fluorescence images of MSCs incubated with DiD-labeled RCNP for different durations (0, 24, and 48 h). RCNP is labeled with DiD (red).

Supplementary Figure 6. Stability of RCNP anchored on the MSC surface. Investigation of the stability of RCNP modification on the MSC surface by assessing the percentage of RCNP bound to MSCs cultured in 10% serum medium over various time interval. Means ± SEM, n =3 independent experiments.

Supplementary Figure 7. Effects of surface engineering on MSC biological functions. a) Representative Live/Dead staining images of MSCs. The images are representative of five samples obtained. Fluorescence imaging was performed immediately after cell staining. Live cells are stained green (Calcein-AM), and dead cells are stained red (PI). b) Representative images showing the migration of native and engineered MSCs. Means ± SEM, n =3 independent experiments. Statistical analysis was performed using one-way ANOVA with Tukey’s multiple-comparisons tests (n.s., not significant). CNP-MSCs, MSCs modified with drug-free neutrophil-mimetic nanoparticles; RCNP-MSCs, MSCs modified with rosiglitazone-loaded neutrophil-mimetic nanoparticles.

Supplementary Figure 8. Gene expression of PPARγ and Cyclin D1 in ROSI-treated MSCs. Means ± SEM, n =3 independent experiments. Statistical analysis was performed by unpaired two-tailed Student’s t-test (**P* < 0.05, ***P*< 0.01). Control, untreated MSCs; ROSI, rosiglitazone-treated MSCs.

Supplementary Figure 9. PCA and KEGG pathway enrichment analysis of ROSI-treated MSCs. (a) Principal component analysis (PCA) of MSCs treated with rosiglitazone (ROSI) or control (native MSCs), showing distinct transcriptional profiles between the two groups. (b) KEGG pathway enrichment analysis of differentially expressed genes between ROSI-treated and control MSCs. Control, untreated MSCs; ROSI, rosiglitazone-treated MSCs.

Supplementary Figure 10. Cell viability of MSCs treated with different concentrations of rosiglitazone. Means ± SEM, n=5 independent experiments.

Supplementary Figure 11. Effect of rosiglitazone treatment on HGF and FGF-2 secretion. Quantification of HGF (left) and FGF-2 (right) levels in the conditioned medium of MSCs treated with rosiglitazone (ROSI) or control. Means ± SEM, n=5 independent experiments. Statistical analysis was performed using Student's t-test (n.s., not significant). Control, untreated MSCs; ROSI, rosiglitazone-treated MSCs.

Supplementary Figure 12. RCNP-MSCs regulate macrophage polarization and associated transcriptional programs. (a) qPCR analysis of the relative mRNA expression levels of the M2-associated markers CD206 and Arg1 and the M1-associated inflammatory genes TNF-α and iNOS in M1 macrophages cultured alone or cocultured with Native MSCs or RCNP-MSCs. (b) qPCR analysis of the relative expression levels of Cd36, IL-6, IL-1b, Stat6, and Klf4 in M1 macrophages cultured alone or cocultured with Native MSCs or RCNP-MSCs. Mean ± SEM, n = 3 independent experiments. Statistical analysis was performed using one-way ANOVA with Tukey’s multiple-comparison test (P < 0.05; P < 0.01; P < 0.001). Native MSCs, unmodified mesenchymal stromal cells; RCNP-MSCs, MSCs modified with rosiglitazone-loaded neutrophil-mimetic nanoparticles.

Supplementary Figure 13. Quantitative immunofluorescence analysis of macrophage polarization markers. Quantification of the mean fluorescence intensity of iNOS and Arg1 in macrophages. Means ± SEM, n=3 independent experiments. Statistical analysis was performed using one-way ANOVA with Tukey’s multiple comparisons tests (***P* < 0.01; ****P* < 0.001; *****P* < 0.0001). RCNP-MSCs, MSCs modified with rosiglitazone-loaded neutrophil-mimetic nanoparticles.

Supplementary Figure 14. Direct and indirect effects of rosiglitazone on MSC apoptosis under M1 macrophage-conditioned inflammatory conditions. (a) Representative Annexin V/PI flow cytometry plots of Native MSCs, Native MSCs cultured with M1 macrophage-conditioned medium (M1-CM), Native MSCs cultured with M1-CM and free ROSI, RCNP-MSCs cultured with M1-CM, Native MSCs cultured with conditioned medium from ROSI-treated M1 macrophages (ROSI-M1-CM), and Native MSCs cultured with conditioned medium generated from M1 macrophages cocultured with RCNP-MSCs (M1/RCNP-MSCs-CM). (b) Quantification of apoptotic MSCs. The percentage of apoptotic MSCs was calculated as the sum of early and late apoptotic cells. Means ± SEM, n=3 independent experiments. Statistical significance was determined using one-way ANOVA followed by Tukey’s multiple-comparison test. (****p* < 0.001, *****p* < 0.0001; ns, not significant). RCNP-MSCs, MSCs modified with rosiglitazone-loaded neutrophil-mimetic nanoparticles.

Supplementary Figure 15. Characterization of DMSO-differentiated HL-60 cells and neutrophil-associated membrane proteins used for nanoparticle coating. (a) Representative flow cytometric profiles of forward scatter (FSC) and quantitative analysis of median FSC values in untreated and DMSO-differentiated HL-60 cells. Mean ± SEM, n = 3 independent experiments. (b) Representative flow cytometric profiles and quantification of CD11b expression in untreated and DMSO-differentiated HL-60 cells. CD11b expression is presented as mean fluorescence intensity (MFI). Mean ± SEM, n = 3 independent experiments. (c) Western blot analysis of CD11b and CXCR2 in membrane proteins derived from untreated or DMSO-differentiated HL-60 cells and used for nanoparticle coating. Statistical analysis in (a) and (b) was performed using an unpaired two-tailed Student’s t-test (*****P* < 0.0001).

Supplementary Figure 16. Representative fluorescence images and quantitative analysis showing the adhesion of MSCs to HUVECs. Means ± SEM, n=3 independent experiments. Statistical analysis was performed using Student's t-test (****P < 0.001*). RCNP-MSCs, MSCs modified with rosiglitazone-loaded neutrophil-mimetic nanoparticles.

Supplementary Figure 17. Toxicity analysis. Representative H&E images depicting different organs obtained from mice within specified groups. The mice were randomly allocated into 5 groups and treated accordingly. After a period of 14 days, the mice were euthanized, and their major organs were excised for H&E staining. Native MSCs, unmodified MSCs; CNP-MSCs, MSCs modified with drug-free neutrophil-mimetic nanoparticles; RCNP-MSCs, RCNP-modified MSCs.

Supplementary Figure 18. Hepatic accumulation of free RCNP in APAP-induced acute liver failure mice. (a) Representative fluorescence images of livers collected from mice administered PBS or DiD-labeled PLGA, RPLGA, or RCNP. (b) Quantification of the total radiant efficiency in the liver. Means ± SEM, n=5 mice. Statistical significance was determined using one-way ANOVA followed by Tukey’s multiple-comparison test (***p* < 0.01). RPLGA, rosiglitazone-loaded PLGA nanoparticles; RCNP, rosiglitazone-loaded neutrophil-mimetic nanoparticles.

Supplementary Figure 19. Biodistribution of MSCs in ALF mice. a) IVIS imaging of the heart, liver, spleen, lungs, and kidneys of mice to observe the distribution of engineered MSCs. b) Quantification of fluorescence intensity in organs from different groups. Means ± SEM, n = 5 mice. Statistical analysis was performed using one-way ANOVA with Tukey’s multiple comparisons tests (****P < 0.0001). RCNP-MSCs, MSCs modified with rosiglitazone-loaded neutrophil-mimetic nanoparticles.

Supplementary Figure 20. Macroscopic liver appearance. Representative photographs of dissected liver from treated or untreated ALF mice after euthanasia. ROSI, free rosiglitazone; Native MSCs, unmodified MSCs; CNP-MSCs, MSCs modified with drug-free neutrophil-mimetic nanoparticles; RCNP-MSCs, MSCs modified with rosiglitazone-loaded neutrophil-mimetic nanoparticles.
